# Association of Survivin Polymorphisms with Tumor Susceptibility: A Meta-Analysis

**DOI:** 10.1371/journal.pone.0074778

**Published:** 2013-09-30

**Authors:** Ying Zhu, Yongguo Li, Shisheng Zhu, Renkuan Tang, Yunzhi Liu, Jianbo Li

**Affiliations:** Department of Forensic Medicine, Faculty of Basic Medical Sciences, Chongqing Medical University, Chongqing, China; National Central University, Taiwan

## Abstract

**Background:**

The survivin polymorphisms have been shown to confer genetic susceptibility to various tumors, but the results are inconsistent. In order to accomplish a more precise estimation of the relationship, a meta-analysis was performed.

**Results:**

For rs9904341, a significantly increased tumor risk was found in overall meta-analysis under C/C vs. G/G (OR = 1.40, 95% CI = 1.13–1.74, p = 0.002), dominant (OR = 1.18, 95% CI = 1.01–1.38, p = 0.039) and recessive (OR = 1.34, 95% CI = 1.13–1.58, p = 0.001) genetic models and Asians group. In subgroup analyses of tumor types, we found a significant association between this SNP and an increased risk of gastric, colorectal, bladder and other tumors as well as a decreased risk of hepatocellular cancer. For rs17878467, a significantly decreased tumor risk was identified in overall meta-analysis for allele contrast (T vs. C: OR = 0.69, 95% CI = 0.51–0.92, p = 0.012), C/T vs. C/C (OR = 0.61, 95% CI = 0.42–0.88, p = 0.009) and dominant (OR = 0.62, 95% CI = 0.43–0.88, p = 0.007) genetic models and Asians group. For rs2071214, we found a significant association between this SNP and an increased tumor risk in overall meta-analysis under G/G vs. A/A (OR = 1.51, 95% CI = 1.04–2.18, p = 0.029) and recessive (OR = 1.54, 95% CI = 1.07–2.22, p = 0.020) genetic models and Asians group. Besides, there was a significant association of rs8073069 with an increased tumor risk under recessive genetic model (OR = 1.37, 95% CI = 1.01–1.84, p = 0.040), while no significant association between rs1042489 and tumor risk was detected.

**Conclusions:**

The survivin rs9904341 most likely contributed to increased susceptibility to tumor in Asians as well as to gastric, colorectal and bladder cancers. As for rs17878467, the T allele might be a protective factor for tumor, especially in Asians. Moreover, the survivin rs8073069 and rs2071214 seemed to be associated with an increased tumor risk in Asians, while there was no association between the survivin rs1042489 and tumor risk.

## Introduction

Apoptosis, also known as programmed cell death, plays an important role in the development and maintenance of tissue homeostasis in multicellular organisms [1], [2]. Apoptosis is orchestrated mainly through the death receptor and mitochondrial pathways leading to a cascade activation of enzymes called caspases [3], [4]. Defects in apoptosis can lead to many human disorders, including tumor [5], [6]. To date, although precise mechanisms that underlie tumorigenesis are not fully understood, inappropriate regulation of apoptosis, owing to recent advancements in tumor biology, are thought to be involved in tumorigenesis via prolonging cell survival, promoting accumulation of transforming mutations and enhancing resistance to therapy [7].

Survivin is a member of the inhibitor of apoptosis protein family, which is involved in the inhibition of apoptosis [8]. Previous evidences have indicated that survivin can block a common downstream part of two major apoptosis pathways, the extrinsic or receptor-mediated pathway and the intrinsic or mitochondrial pathway, through directly and/or indirectly inhibiting initiator (caspase-9) and effector caspases (caspase-3 and -7), and thus preventing apoptosis [9]. Recently, increased level of survivin expression has been found in various malignancies, including gastric, colorectal and other tumors [10], [11], [12], [13], suggesting that survivin may play a critical role in tumorigenesis, with important biological, prognostic and therapeutic implications.

**Figure 1:**
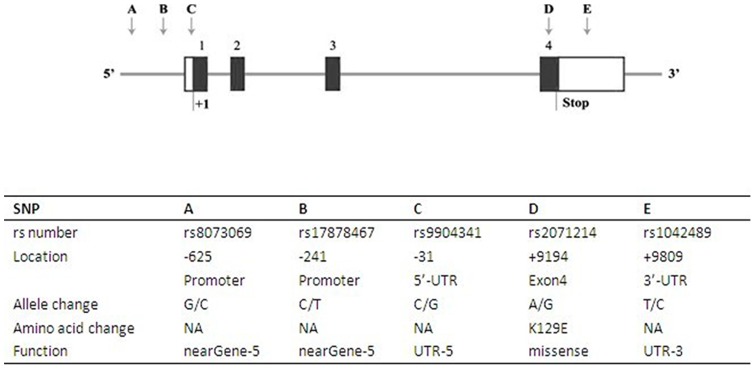
The structure of survivin gene and the features of SNPs in survivin gene that analyzed in this meta-analysis. Exons are shown by the black box and are numbered 1, 2, 3, and 4 from the 5′- to 3′-end of the gene; introns are shown by the thin line; the untranslated portions of the gene are shown by the white box; the SNPs in survivin gene are shown by the arrow and labeled A to E. The start and stop sites of transcript are shown by “+1” and “Stop”, respectively.

Survivin is expressed in a cell-cycle-regulated manner, with a peak in the G2/M phase of the cell cycle, which is largely controlled at the genetic level [14]. The human survivin gene is encoded by baculoviral inhibitor of apoptosis repeat-containing 5 (BIRC5) and consists of four exons spanning 14.7 kb at the telomeric position of chromosome 17q25 (Figure 1) [15]. It is well known that gene expression can be influenced by a single nucleotide polymorphism (SNP) located within the promoter and/or other regulatory regions of the gene. Therefore, polymorphisms of survivin gene may have a functional consequence affecting the production or activity of survivin, thus regulating the individual's susceptibility to tumors.

Many studies have recently explored the potential association of several SNPs in survivin gene with the susceptibility to various tumors [16], [17], [18], [19], [20], [21], including -625G/C (rs8073069), -241C/T (rs17878467) and -31G/C (rs9904341) located in the promoter region as well as +9194A/G (rs2071214) and +9809C/T (rs1042489) respectively found in exon4 and 3′-UTR (Figure 1). However, the observed associations of these studies are inconsistent and each of these trials has not been large enough to detect the effect of these survivin SNPs on tumor risk. As a result, we performed a meta-analysis of all eligible studies to derive more precise estimation of the association of the above-mentioned survivin SNPs with tumor risk.

## Materials and Methods

### Protocols and Eligibility Criteria

The present meta-analysis and systematic review follows the Preferred Reporting Items for Systematic Review and Meta-analyses (PRISMA) statements (Checklist S1). And the focused question is adapted by using the Population, Intervention, Comparison, Outcomes (PICO) criteria. The literature search was limited to original studies performed in humans on the association of the above-mentioned survivin SNPs with tumor risk. There was no publication year restriction applied.

### Search strategy

PubMed, EMBASE, and Chinese National Knowledge Infrastructure (CNKI) were searched till the end of January 2013 to identify relevant studies. Literature searching was performed by applying combinations of the following terms: (“survivin” or “Baculoviral inhibitor of apoptosis repeat-containing 5” or “BIRC5”) and (“genetic variant” or “genetic variation” or “polymorphism”) and (“tumor” or “cancer” or “carcinoma” or “neoplasia” or “neoplasms”). These keywords were used as MESH headings and free text words. Besides, the manual searching of reference lists from potentially relevant papers was also performed, based on the computer-assisted strategy, to identify any additional studies that may have been missed.

### Selection of studies

The inclusive criteria were: (1) studies used validated genotyping methods (for example, PCR-RFLP) to measure the association of survivin polymorphisms with tumor risk; (2) studies were in an appropriate analytical design (such as case-control, cohort, or nested case control); (3) studies were published in English or Chinese; (4) studies with the full-text availability; (5) data were not duplicated in another manuscript. Studies were excluded if they did not report the relevant data to calculate the odds ratios and its variance. In addition, studies were also excluded if control subjects in these studies departed from Hardy-Weinberg equilibrium (HWE).

### Data extraction

Two reviewers (ZY and ZSS) independently performed the data extraction, with the disagreements resolved through consensus decision. For each trial, the following items were collected: the surname of first author, year of publication, country, ethnicity (categorized as Caucasian, Asian or Mixed (the original study did not state the detailed ethnic result of subjects or mixed races)), sample size, types of tumors, matching criteria of cases and controls, control source, genotyping methods, genes and variants genotyped.

### Statistical analysis

The evidence of HWE in controls was recalculated in the present meta-analysis through the application of the online software (http://ihg2.helmholtz-muenchen.de/cgi-bin/hw/hwa1.pl). And a p–value less than 0.05 of HWE was considered significant.

Meta-analysis for a certain SNP of survivin gene was conducted by using the software Stata, version 11.0 (Stata Corp., College Station, TX) when data were available from more than three studies. The strength of association between survivin polymorphisms and tumor risk was measured by odds ratios (ORs) with their 95% confidence intervals (CIs). The significance of pooled ORs was determined by the Z–test, and statistical significance was defined as a 2-sided P-value <0.05. As for survivin rs9904341, allele frequency comparison model (C versus G) was initially used to examine the potential association of the assumed risk allele C with tumor susceptibility, then different comparison models, including additive (C/C versus G/G and G/C versus G/G), dominant (C/C+G/C versus G/G), and recessive (C/C versus G/C+G/G) genetic models, were also used to estimate tumor risk, as well as other SNPs of survivin gene. Besides, subgroup analyses were stratified by ethnicity and types of tumors, respectively.

A fixed-effects model (the Mantel–Haenszel method) was firstly used to estimate the pooled ORs, while the random-effects model (the DerSimonian and Laird method) was planned to be used if there was evidence of significant heterogeneity across trials (P_h_<0.1 and *I^2^>*50%). A sensitivity analysis was performed to explore the potential source of heterogeneity. In addition, publication bias was assessed by visual inspection of funnel plots in which the standard error of log (OR) of each study was plotted against its log (OR). Funnel plot asymmetry was assessed by Egger's linear regression test.

## Results

### Literature search

**Figure 2:**
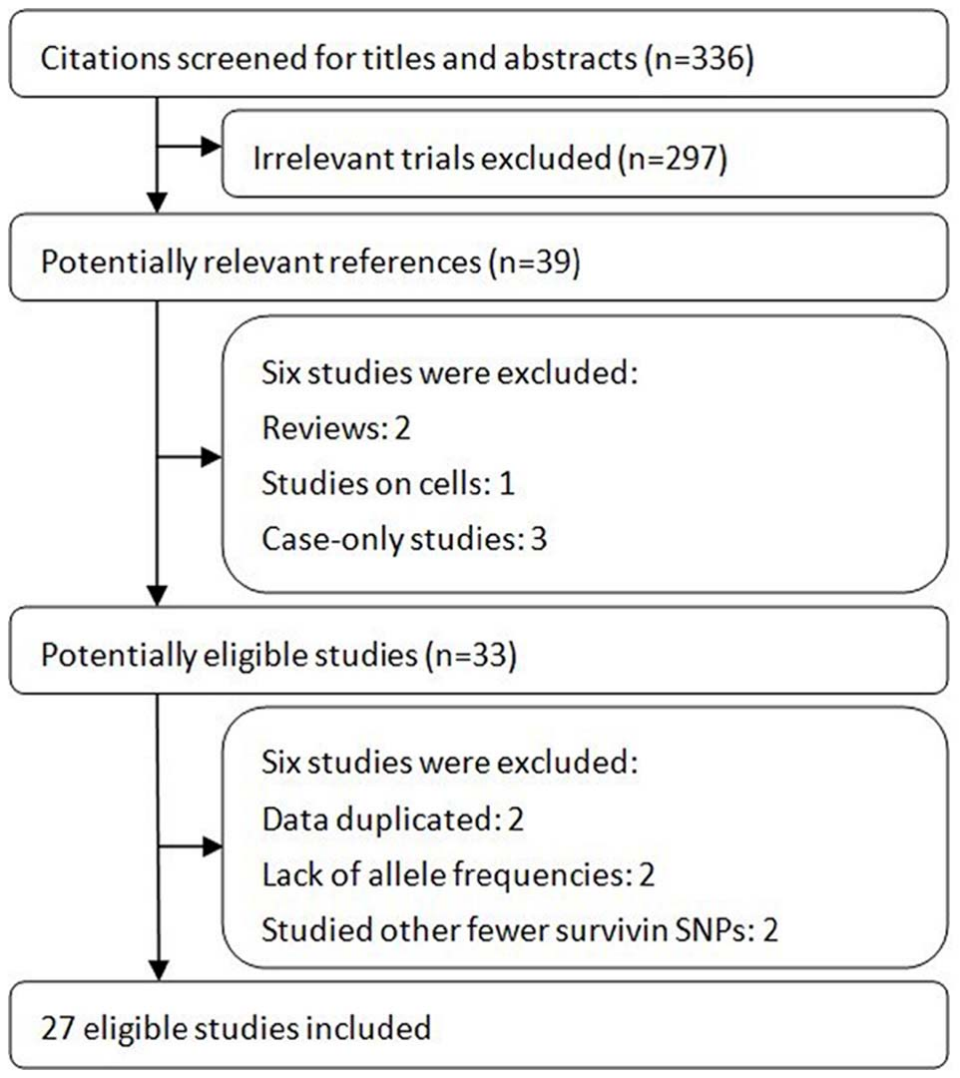
Flow of study identification, inclusion, exclusion.

Figure 2 shows details of study identification, inclusion, and exclusion. The literature search under the defined terms yielded 336 articles. By screening the titles and abstracts, 297 articles were excluded due to the irrelevance to this topic. In 39 potentially relevant references, 33 articles were taken for a comprehensive evaluation. After a full retrieval of all these articles, two Chinese studies were excluded which had data duplicated [22], [23], another two studies were excluded because they did not report allele frequencies used for calculating ORs and 95% CIs [24], [25], and other two studies were also excluded because they studied fewer survivin SPNs (e.g. rs2239680, rs1042542) [26], [27]. Finally, twenty seven studies involving a total of 6,468 cases and 7,983 controls were included in this meta-analysis.

### Studies characteristics

The main characteristics of included studies are shown in Table 1. As for survivin rs9904341, the ultimate analysis included four gastric cancer studies [28], [29], [30], [31], three hepatocellular cancer studies [20], [32], [33], three colorectal cancer studies [34], [35], [36], two esophageal [18], [37] and two bladder cancer studies [19], [21], as well as twelve studies of other tumors [16], [17], [38], [39], [40], [41], [42], [43], [44], [45], [46], [47]. Overall, 8 studies used Caucasians, 17 used Asians, one used mixed populations. With regard to survivin rs2071214, five studies that comprised a total of 1512 cases and 1990 controls were used for the final analysis [17], [19], [20], [45], [48]. Among these studies, one used Caucasians and four used Asians. For survivin rs17878467, the final analysis included three studies that used Asians [20], [21], [45] and one that used Caucasians [46]. Besides, there were three studies [17], [18], [33] and four studies [17], [20], [33], [45] that used Asians to investigate rs8073069 and rs1042489, respectively. Moreover, the genotype distributions among the controls of all included studies were consistent with HWE (Table 1).

**Table 1:** Main Characteristics of Included Studies.

Author, year (country)	Ethnicity	Sample size (case/control)	Types of tumor	Matching criteria	control source	Genotype method	Polymorphism	HWE in controls				
								rs8073069	rs17878467	rs9904341	rs2071214	rs1042489
**Borbely, 2007 Hungary**	Caucasian	81/180	Cervical	_	Population based	PCR-RFLP	rs9904341			0.856		
**Jang, 2008 Korea**	Asian	582/582	Lung	Age, gender	Hospital based	FLH	rs8073069 rs9904341 rs2071214 rs1042489	0.494		0.867	0.664	0.563
**Cheng, 2008 China**	Asian	96/67	Gastric	Age	Population based	RT-PCR	rs9904341			0.667		
**Wang, 2009 Taiwan, China**	Asian	190/210	Urothelial	Age, gender	Hospital based	PCR-RFLP	rs9904341			0.231		
**Gazouli, 2009 Greece**	Caucasian	312/362	Colorectal	_	Hospital based	PCR-RFLP	rs9904341			0.110		
**Yang, 2009 China**	Asian	220/220	Gastric	Age, gender	Hospital based	PCR-RFLP	rs9904341			0.104		
**Huang, 2009 China**	Asian	702/711	Colorectal	Age, gender	Hospital based	PCR-RFLP	rs9904341			0.432		
**Yang, 2009 China**	Asian	221/268	Esophageal	Age, gender	Hospital based	PCR-RFLP	rs8073069 rs9904341	0.455		0.249		
**Kawata, 2010 Japan**	Asian	235/346	Bladder	_	Hospital based	PCR-RFLP	rs9904341 rs2071214			0.228	0.167	
**Borges, 2010 Brazil**	Mixed	47/57	Gastric	_	Hospital based	Quantitative PCR, Sequencing	rs9904341			0.784		
**Theodoropoulos, 2010 Greece**	Caucasian	80/160	Pancreatic	Age, gender	Population based	PCR-RFLP	rs9904341			0.062		
**Upadhyay, 2010 India**	Asian	250/250	Esophageal	Age, gender	Hospital based	PCR-RFLP	rs9904341			0.094		
**Antonacopoulou, 2011 Greece**	Caucasian	163/132	Colorectal	_	Population based	RT-PCR	rs9904341			0.184		
**Ma, 2011 China**	Asian	855/1036	Nasopharyngeal	Age, gender	Hospital based	TaqMan	rs9904341			0.357		
**Bayram, 2011 Turkey**	Caucasian	160/241	Hepatocellular	Age, gender, smoking and alcohol consumption	Hospital based	PCR-RFLP	rs9904341			0.109		
**Ulybina, 2011 Russia**	Caucasian	121/142	breast	_	Population based	_	rs2071214				0.631	
**Zahedi, 2012 Iran**	Asian	31/30	Endometrial	_	Hospital based	PCR-RFLP	rs9904341			0.524		
**Hsieh, 2012 Taiwan, China**	Asian	135/496	Hepatocellular	Race, ethnicity	Hospital based	TaqMan	rs17878467 rs9904341 rs2071214 rs1042489		1.000	0.663	0.307	0.788
**Li, 2012 China**	Asian	178/196	Hepatocellular	Age, gender	Population based	PCR-RFLP	rs8073069 rs9904341 rs1042489	0.706		0.779		0.228
**Qin, 2012 China**	Asian	710/760	Renal	Age, gender	Hospital based	TaqMan	rs9904341			0.610		
**Jaiswal, 2012 Indian**	Asian	200/200	Bladder	Age, gender	Hospital based	PCR-RFLP	rs17878467 rs9904341		0.120	0.471		
**Yazdani, 2012 Iran**	Asian	123/131	Papillary thyroid	_	Population based	PCR-RFLP	rs9904341			0.407		
**Andric, 2012 Serbia**	Caucasian	52/82	Keratocystic odontogenic	Age, gender	Hospital based	PCR-RFLP	rs9904341			0.220		
**Weng, 2012 Taiwan, China**	Asian	439/424	Oral	Race	Hospital based	TaqMan	rs17878467 rs9904341 rs2071214 rs1042489		1.000	0.507	0.197	0.698
**Radojevic-Skodric, 2012 Serbia**	Caucasian	59/82	Wilms	Ethnicity	Population based	PCR-RFLP	rs17878467 rs9904341		0.228	0.220		
**Jin, 2012** **China**	Asian	138/138	Ovarian	_	Hospital based	PCR-LDR	rs9904341			0.505		
**Liarmakopoulos, 2012 Greece**	Caucasian	88/480	Gastric	Age, gender	Hospital based	PCR-RFLP	rs9904341			0.063		

PCR: polymerase chain reaction; RFLP: restriction fragment length polymorphism; FLH: fluorescence labeled hybridization; RT: reverse transcription; LDR: ligase detection reaction; HWE: Hardy-Weinberg equilibrium.

A p–value less than 0.05 of HWE was considered significant.

### Meta-analysis results

**Figure 3:**
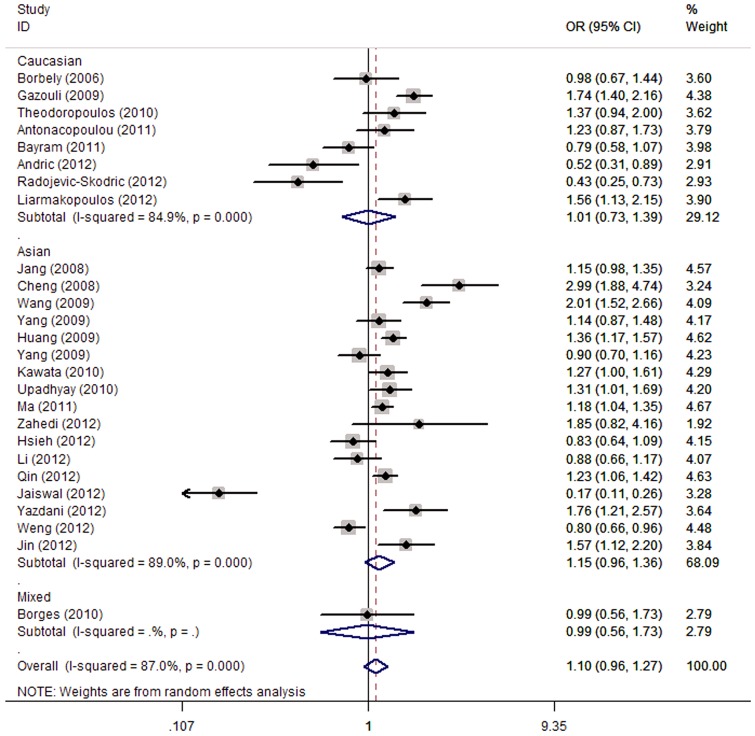
Forest plot of tumor risk associated with the survivin rs9904341 under the allele contrast.

For the survivin rs9904341, a significantly increased tumor risk was found for C/C vs. G/G (OR = 1.40, 95% CI = 1.13–1.74, p = 0.002), dominant (OR = 1.18, 95% CI = 1.01–1.38, p = 0.039) and recessive (OR = 1.34, 95% CI = 1.13–1.58, p = 0.001) genetic models, while there was no significant association of this SNP with tumor risk under allele frequency comparison model and C/C vs. G/G genetic model (Table 2, Figure 3). In the stratified analysis by ethnicity, we found a significant association of this SNP with an increased tumor risk in Asians under C/C vs. G/G (OR = 1.52, 95% CI = 1.19–1.95, p = 0.001), dominant (OR = 1.25, 95% CI = 1.06–1.47, p = 0.006) and recessive (OR = 1.40, 95% CI = 1.16–1.70, p = 0.001) genetic models; however, no evidence of associations was detected in Caucasian and mixed populations (Table 2). In order to better understand the association of this SNP with susceptibility of various tumors, we also performed a subgroup analysis according to the type of tumors. The overall ORs with its 95% CI showed a statistical association between this SNP and an increased risk of gastric cancer (C/C vs. G/G: OR = 2.21, 95% CI = 1.06–4.64, p = 0.035; recessive genetic model: OR = 1.85, 95% CI = 1.12–3.04, p = 0.016), bladder cancer (C/C vs. G/G: OR = 1.76, 95% CI = 1.20–2.59, p = 0.004; recessive genetic model: OR = 1.86, 95% CI = 1.36–2.54, p = 0.001) and other tumors (recessive genetic model: OR = 1.29, 95% CI = 1.02–1.62, p = 0.035) (Table 2). In addition, all five genetic models produced a significant association of this SNP with an increased risk of colorectal cancer, while there was no significant association between this SNP and esophageal cancer risk under all five genetic models (Table 2). Surprisingly, pooled ORs revealed a significantly decreased risk of hepatocellular cancer under the allele frequency comparison model (C vs. G: OR = 1.10, 95% CI = 0.96–1.27, p = 0.180), but other genetic models did not reveal such an association (Table 2). As a result, more convincing evidence, such as larger sample size and number of studies, is required to draw a more solid conclusion of the association of this SNP with hepatocellular cancer risk.

**Table 2:** Meta-analysis results of *survivin* polymorphisms and tumor risk.

*Survivin* rs8073069	C vs. G OR (95% CI), P P_h_, *I^2^*(%); P_E_	C/C vs. G/G OR (95% CI), PP_h_, *I^2^*(%); P_E_	G/C vs. G/G OR (95% CI), PP_h_, *I^2^*(%); P_E_	Dominant genetic model OR (95% CI), PP_h_, *I^2^*(%); P_E_	Recessive genetic model OR (95% CI), PP_h_, *I^2^*(%); P_E_
**Total/Ethnicity (Asian)**	1.12 (0.85–1.47), 0.433 0.028, 72.1; 0.670	1.46 (0.82–2.61), 0.195 0.053, 66.0; 0.613	0.96 (0.80–1.15), 0.652 0.235, 31.0; 0.755	1.05 (0.78–1.43), 0.731 0.080, 60.5; 0.657	**1.37 (1.01–1.84), 0.040 0.120, 52.7; 0.592**
***Survivin*** **rs17878467**	**T vs. COR (95% CI), P P_h_, ** ***I^2^*** **(%); P_E_**	**T/T vs. C/COR (95% CI), P** **P_h_, ** ***I^2^*** **(%); P_E_**	**C/T vs. C/COR (95% CI), P** **P_h_, ** ***I^2^*** **(%); P_E_**	**Dominant genetic modelOR (95% CI), P** **P_h_, ** ***I^2^*** **(%); P_E_**	**Recessive genetic model OR (95% CI), P** **P_h_, ** ***I^2^*** **(%); P_E_**
**Total**	**0.69 (0.51–0.92), 0.012 0.639, 0.0; 0.153**	0.74 (0.38–1.46), 0.389 0.563, 0.0; 0.149	**0.61 (0.42–0.88), 0.009 0.606, 0.0; 0.237**	**0.62 (0.43–0.88), 0.007 0.629, 0.0; 0.173**	0.86 (0.44–1.68), 0.663 0.578, 0.0; 0.207
**Ethnicity**					
**Caucasian**	0.75 (0.43–1.30), 0.303 NA	1.11 (0.26–4.75), 0.890 NA	0.54 (0.26–1.10), 0.091 NA	0.59 (0.30–1.18), 0.135 NA	1.42(0.34–5.92), 0.632 NA
**Asian**	**0.66 (0.47–0.94), 0.020 0.457, 0.0; 0.359**	0.67 (0.31–1.43), 0.299 0.428, 0.0; 0.344	**0.64 (0.41–0.99), 0.043 0.431, 0.0; 0.346**	**0.62 (0.41–0.94), 0.024 0.423, 0.0; 0.341**	0.75 (0.35–1.60), 0.460 0.495, 0.0; 0.380
***Survivin*** **rs9904341**	**C vs. GOR (95% CI), PP_h_, ** ***I^2^*** **(%); P_E_**	**C/C vs. G/GOR (95% CI), PP_h_, ** ***I^2^*** **(%); P_E_**	**G/C vs. G/GOR (95% CI), PP_h_, ** ***I^2^*** **(%); P_E_**	**Dominant genetic modelOR (95% CI), PP_h_, ** ***I^2^*** **(%); P_E_**	**Recessive genetic model OR (95% CI), PP_h_, ** ***I^2^*** **(%); P_E_**
**Total**	1.10 (0.96–1.27), 0.1800.001, 87.0; 0.380	**1.40 (1.13–1.74), 0.0020.001, 75.0; 0.826**	1.08 (0.94–1.25), 0.2690.001, 62.8; 0.608	**1.18 (1.01–1.38), 0.0390.001, 73.2; 0.822**	**1.34 (1.13–1.58), 0.0010.001, 69.8; 0.673**
**Ethnicity**					
**Caucasian**	1.01 (0.73–1.39), 0.9630.001, 84.9; 0.096	1.04 (0.60–1.82), 0.8790.001, 76.5; 0.061	0.95 (0.62–1.44), 0.7930.001, 79.2; 0.173	0.98 (0.63–1.53), 0.9280.001, 83.8; 0.117	1.09 (0.73–1.64), 0.6640.006, 64.8; 0.101
**Asian**	1.15 (0.96–1.36), 0.1220.001, 89.0; 0.856	**1.52 (1.19–1.95), 0.0010.001, 77.2; 0.318**	1.11 (0.97–1.28), 0.1280.009, 50.8; 0.130	**1.25 (1.06–1.47), 0.0060.001, 67.3; 0.086**	**1.40 (1.16–1.70), 0.0010.001, 74.5; 0.543**
**Mixed**	0.99 (0.56–1.73), 0.965 NA	1.18 (0.38–3.67), 0.773 NA	0.68 (0.29–1.58), 0.366 NA	0.79 (0.36–1.74), 0.553 NA	1.45 (0.51–4.11), 0.484 NA
**Types of tumor**					
**Gastric**	1.51 (0.99–2.30), 0.056 0.002, 79.5; 0.637	**2.21 (1.06–4.64), 0.0350.013, 72.4; 0.576**	1.26 (0.77–2.07), 0.3630.068, 57.9; 0.961	1.52 (0.85–2.73), 0.1590.011, 73.1; 0.782	**1.85 (1.12–3.04), 0.0160.068, 57.9; 0.416**
**Hepatocellular**	**0.83 (0.71–0.98), 0.0320.886, 0.0; 0.673**	0.70 (0.49–1.00), 0.0500.943, 0.0; 0.526	0.86 (0.66–1.12), 0.2580.366, 0.6; 0.321	0.81 (0.63–1.04), 0.0990.528, 0.0; 0.237	0.75 (0.56–1.01), 0.0610.842, 0.0; 0.789
**Colorectal**	**1.44 (1.28–1.61), 0.0010.115, 53.8; 0.991**	**1.83 (1.19–2.82), 0.0060.067, 63.0; 0.805**	**1.28 (1.05–1.56), 0.0140.186, 40.6; 0.084**	**1.49(1.24–1.80), 0.0010.282, 21.0; 0.529**	**1.58 (1.07–2.32), 0.0200.048, 67.0; 0.630**
**Esophageal**	1.08 (0.75–1.56), 0.6730.041, 75.9; NA	1.32 (0.51–3.46), 0.5680.012, 84.2; NA	0.99 (0.74–1.32), 0.9250.947, 0.0; NA	1.06 (0.80–1.38), 0.6960.421, 0.0; NA	1.32 (0.50–3.50), 0.5710.004, 87.9; NA
**Bladder**	0.47 (0.06–3.43), 0.4540.001, 98.4; NA	**1.76 (1.20–2.59), 0.0040.207, 37.1; NA**	0.97 (0.72–1.31), 0.8530.245, 26.0; NA	1.18 (0.89–1.57), 0.2480.332, 0.0; NA	**1.86 (1.36–2.54), 0.0010.408, 0.0; NA**
**Other**	1.15 (0.95–1.37), 0.1450.001, 82.6; 0.881	1.32 (0.94–1.85), 0.1040.001, 77.5; 0.810	1.07 (0.83–1.39), 0.5940.001, 76.0; 0.800	1.15 (0.87–1.51), 0.3300.001, 81.2; 0.921	**1.29 (1.02–1.62), 0.0350.001, 66.5; 0.665**
***Survivin*** **rs2071214**	**G vs. AOR (95% CI), P** **P_h_, ** ***I^2^*** **(%); P_E_**	**G/G vs. A/AOR (95% CI), P** **P_h_, ** ***I^2^*** **(%); P_E_**	**A/G vs. A/AOR (95% CI), P** **P_h_, ** ***I^2^*** **(%); P_E_**	**Dominant genetic modelOR (95% CI), P** **P_h_, ** ***I^2^*** **(%); P_E_**	**Recessive genetic model OR (95% CI), P** **P_h_, ** ***I^2^*** **(%); P_E_**
**Total**	1.05 (0.84–1.31), 0.6830.042, 59.7; 0.575	**1.51 (1.04–2.18), 0.0290.189, 34.9; 0.978**	0.97 (0.83–1.13), 0.652 0.105, 47.8; 0.408	0.99 (0.78–1.27), 0.963 0.060, 55.8; 0.481	**1.54 (1.07–2.22), 0.020 0.288, 19.9; 0.962**
**Ethnicity**					
**Caucasian**	0.63 (0.23–1.73), 0.371 NA	1.13 (0.07–18.26), 0.932 NA	0.41 (0.13–1.32), 0.136 NA	0.51 (0.17–1.52), 0.229 NA	1.18 (0.07–19.12), 0.906 NA
**Asian**	1.07 (0.85–1.35), 0.553 0.031, 66.1; 0.878	**1.52 (1.05–2.20), 0.028 0.107, 50.8; 0.935**	0.98 (0.84–1.15), 0.820 0.135, 46.1; 0.931	1.03 (0.80–1.31), 0.835 0.058, 59.9; 0.867	**1.55 (1.07–2.24), 0.020 0.175, 39.5; 0.953**
***Survivin*** **rs1042489**	**T vs. COR (95% CI), P P_h_, ** ***I^2^*** **(%); P_E_**	**T/T vs. C/COR (95% CI), P P_h_, ** ***I^2^*** **(%); P_E_**	**C/T vs. C/COR (95% CI), P P_h_, ** ***I^2^*** **(%); P_E_**	**Dominant genetic modelOR (95% CI), P** **P_h_, ** ***I^2^*** **(%); P_E_**	**Recessive genetic model OR (95% CI), P** **P_h_, ** ***I^2^*** **(%); P_E_**
**Total/Ethnicity (Asian)**	1.12 (0.88–1.42), 0.365 0.002, 79.1; 0.887	1.24 (0.76–2.03), 0.389 0.003, 78.3; 0.873	1.11 (0.91–1.35), 0.326 0.140, 45.2; 0.810	1.17 (0.82–1.67), 0.385 0.024, 68.3; 0.996	1.15 (0.84–1.57), 0.381 0.016, 70.8; 0.752

OR: odds ratio; CI: confidence interval; P_h_: the P-value of heterogeneity; P_E_: the P-value of Egger's test; NA: not applicable. When P_h_ is <0.1 and *I^2^* exceeds 50%, the random-effects model is used. Conversely, the fixed-effects model is used.

**Figure 4:**
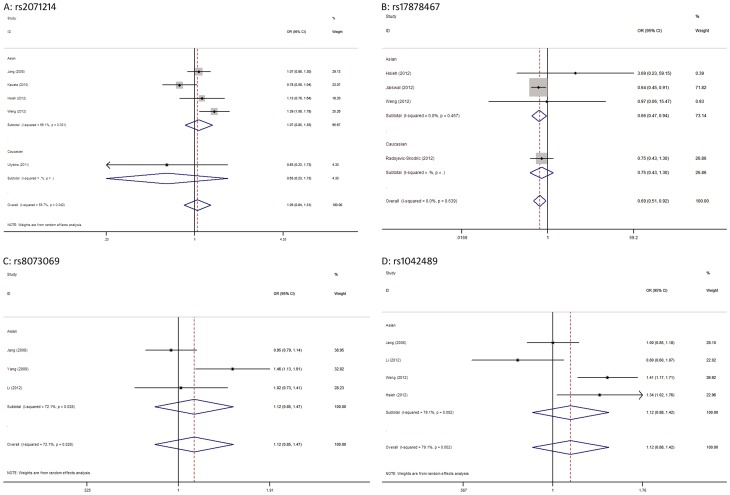
Forest plots of the association of other survivin SNPs with tumor risk under the allele contrast. A: rs2071214, B: rs17878467, C: rs8073069, D: rs1042489.

For the survivin rs2071214 (Table 2, Figure 4A), we found a significant association between this SNP and an increased tumor risk under G/G vs. A/A (OR = 1.51, 95% CI = 1.04–2.18, p = 0.029) and recessive (OR = 1.54, 95% CI = 1.07–2.22, p = 0.020) genetic models. In ethnicity subgroup analysis, this SNP proved to be associated with an increased tumor risk in Asians (G/G vs. A/A: OR = 1.52, 95% CI = 1.05–2.20, p = 0.028; recessive genetic model: OR = 1.55, 95% CI = 1.07–2.24, p = 0.020), but not in Caucasians (Table 2). For the survivin rs17878467 (Table 2, Figure 4B), a significantly decreased tumor risk was identified for allele contrast (T vs. C: OR = 0.69, 95% CI = 0.51–0.92, p = 0.012), C/T vs. C/C (OR = 0.61, 95% CI = 0.42–0.88, p = 0.009) and dominant (OR = 0.62, 95% CI = 0.43–0.88, p = 0.007) genetic models, these results were robust and there was no evidence of heterogeneity across the trials. Subgroup analysis stratified by ethnicity found a significant association of this SNP with a decreased tumor risk in Asians (T vs. C: OR = 0.66, 95% CI = 0.47–0.94, p = 0.020; C/T vs. C/C: OR = 0.64, 95% CI = 0.41–0.99, p = 0.043; dominant genetic model: OR = 0.62, 95% CI = 0.41–0.94, p = 0.024), but not in Caucasians (Table 2).

For the survivin rs8073069 and rs1042489 (Table 2, Figure 4C, D), there was a significant association of the former SNP with an increased tumor risk in recessive genetic model (OR = 1.37, 95% CI = 1.01–1.84, p = 0.040), while no significant association between the latter SNP and tumor risk was detected under all five genetic models. Due to a limited number of included studies reporting these two SNPs, no further subgroup analyses were performed.

### Sensitivity analysis

In order to assess the robustness of our results, we performed the sensitivity analysis with each study removed for every meta-analysis. The results of sensitivity analyses indicated that pooled ORs before and after exclusion of the study which majorly contributed to heterogeneity were generally similar, suggesting that most evidences from our meta-analysis should be considered to be stable and convincing (Table S1).

### Publication Bias

**Figure 5:**
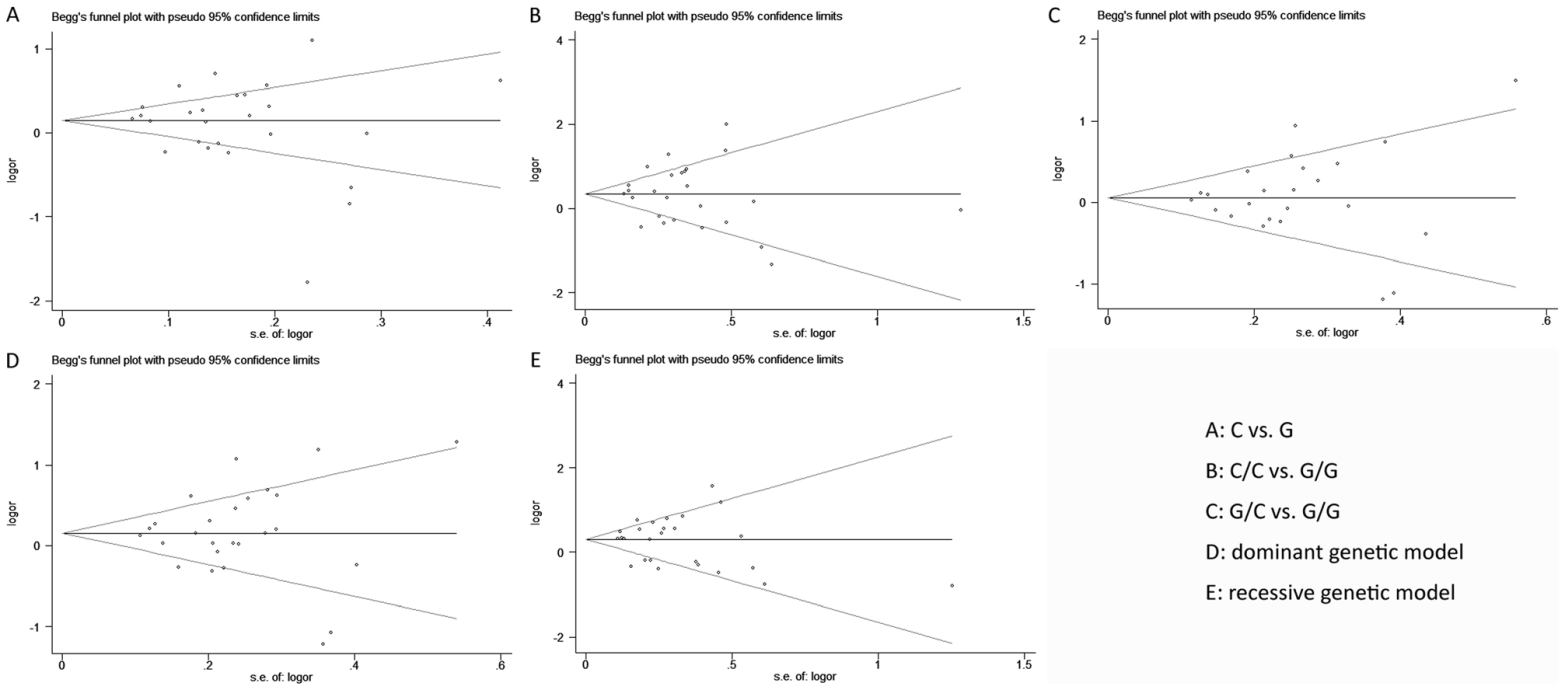
Begg's funnel plot of the survivin rs9904341 and tumor risk in different contrast models. A: C vs. G, B: C/C vs. G/G, C: G/C vs. G/G, D: dominant genetic model (C/C+G/C vs. G/G), and E: recessive genetic model (C/C vs. G/C+G/G).

Publication bias of the included trials was assessed by Begg's funnel plot and Egger's test. As for survivin rs9904341, symmetrical funnel plots were obtained under all genetic models (Figure 5), and the results of Egger's test also suggested no publication bias in these meta-analyses (Table 2). Similarly, no publication bias was detected for association of other survivin polymorphisms with tumor risk (Table 2).

## Discussion

It is generally considered that tumor is a multifactorial disease involving both environmental and genetic factors; however, the precise molecular mechanism of which still remains unclear [49]. Enhancing our understandings of the molecular biology of tumor will help to clarify pathogenesis of this multifactorial disease and to potentially improve patients' clinical outcomes. A wide range of evidences have demonstrated that the regulation of apoptosis is important for the prevention of tumorigenesis. And impairment of apoptosis may cause the accumulation of genetic errors through prolongation of cell cycle, promotion of resistance to immune-based cytotoxicity and a selective growth advantage for the altered cells contributing to tumorigenesis [50]. Survivin (also called baculoviral IAP repeat-containing protein 5, BIRC5) is an anti-apoptotic protein that is implicated both in the regulation of cell cycle and in the inhibition of apoptosis [8], [15]. Previous studies have revealed that survivin is abundantly expressed in embryonic and fetal tissues but is almost undetectable in most terminally differentiated normal tissues [13]. In contrast, obvious over-expression of survivin is commonly observed in a variety of tumors [51]. Furthermore, a positive correlation between the expression of survivin and tumor grade as well as recurrence has also been found [50]. Based on these facts, survivin is considered to be one of the most promising diagnostic and prognostic markers in monitoring tumors.

Growing evidences have suggested that several factors including genetic variation of suvivin gene can modulate the expression of survivin, especially the functional SNPs [13]. And over-expression of survivin that probably resulted from the higher production genotype of these SNPs in survivin gene may provide the molecular bases for a decreased apoptotic capacity to eliminate cells with DNA damage, thus leading to increased susceptibility to tumor. However, studies investigating the potential association of these SNPs in survivin gene with tumor risk have not yielded consistent results. Several studies have supported that risk for tumor is associated with these survivin SNPs [17], [19], [36], [38], while others have failed to find such an association [16], [32], [33], [48]. Therefore, we performed the present meta-analysis to clarify the relationship between these survivin SNPs and tumor risk. Overall, pooled ORs showed that a significant association of survivin rs9904341, rs2071214, rs17878467 and rs8073069 with tumor risk, while there was no significant association between survivin rs1042489 and susceptibility to tumor.

The survivin rs9904341 is identified in the survivin promoter, which is located at the cell cycle-dependent elements (CDEs) and cell cycle homology regions (CHRs) repressor binding site. In-vitro analyses have shown that this mutation can derepress the cell cycle-dependent transcription of survivin gene through the functional disruption of binding at the CDE/CHR repressor motifs and result in over-expression of survivin at both mRNA and protein levels [52]. Besides, functional studies have revealed that the C allele of this SNP has significantly higher transcriptional activity compared to the G allele, and individuals carrying the CC genotype have up-regulated survivin levels than those carrying the GC and GG genotypes [17]. Moreover, two recent meta-analysis of the association between this SNP and tumor risk have indicated that the variant genotypes are associated with a significantly increased tumor risk, and in the stratified analysis by ethnicity, significantly increased tumor risk is associated with Asians, while no significant association is observed in the subgroup analysis of cancer type [53], [54].

Consistent with the functional studies [17], our results suggested that the survivin rs9904341 was associated with a significantly increased tumor risk under several genetic models. In the subgroup analysis stratified by ethnicity, our results revealed a significant association of this SNP with an increased tumor risk only in Asians but not in Caucasian and mixed populations, which were also consistent with the two prior meta-analyses, indicating genetic heterogeneity between different ethnicities. In the subgroup analysis of cancer type, we found a significant association between this SNP and an increased risk of gastric cancer, colorectal cancer, bladder cancer and other tumors and a decreased risk of hepatocellular cancer, while no significant association of this SNP with risk for esophageal cancer was detected. Our results of this subgroup analysis were not completely consistent with the two prior meta-analyses. One possible reason for this discrepancy could be that our meta-analysis of this SNP included more studies with a much larger sample size to investigate its associations with risk of several tumors. For example, in the subgroup analysis of gastric cancer, we included one more study with 88 cases and 480 controls for the risk association with survivin rs9904341 [31], leading to a sample size of more than 500 subjects, which increased the weight of gastric cancer and study power. Moreover, we found a significant association between this SNP and an increased risk of gastric cancer.

Although the survivin rs17878467 and rs8073069 are also positioned in the survivin promoter, they are not cis-acting element or located in putative transcription factor binding site. However, like survivin rs9904341, there was a significant association of these two SNPs with tumor risk. Consistent with the previous studies [21], we found a significantly protective effect of survivin C variant in rs17878467 for tumor. Besides, the T allele in rs17878467 might also be a protective factor in Asians. As for survivin rs8073069, a significant association of this SNP with an increased tumor risk was only detected under the recessive genetic model, indicating that this SNP might have a small effect on tumor risk. Our results of this SNP were consistent with the study by Yang et al. [18], but inconsistent with the study by Jang et al. [17] and Li et al. [33]. This discrepancy may be explained by the fact that genetic susceptibility is often different in diverse tumors; other molecular and cellular mechanisms are probably involved in over-expression of survivin in these tumors. Overall, the above three mentioned SNPs in the survivin promoter may potentially alter the transcription activity of survivin gene and may have some functional relevance and thus affect individuals' susceptibility to tumors. However, further studies with lager samples are still needed to validate these positive findings.

The survivin rs2071214 leads to amino acid change from Lys to Glu at codon 129 in exon 4, which is located at the C-terminal end of the protein (142 amino acids). Pooled ORs showed that individuals with the +9194GG genotype had an increased risk of tumor when compared to AA or AA/AG genotypes. Our results were consistent with the previous studies [17], [45], which also found a strong linkage disequilibrium between this SNP and survivin rs9904341. As the biological role of amino acid alterations associated with this SNP has not yet been clarified, the role of this SNP in the development of tumor among different racial groups still requires further investigation. Besides, the survivin rs1042489 is located at the 3′-UTR of the survivin gene, regulatory events such as mRNA stability and post-transcriptional modification may occur through binding of microRNAs. Our result showed that this SNP did not correlate with the risk of tumor, suggesting that this SNP probably had nothing to do with the stability of survivin mRNA or its translational efficiency [55]. However, additional studies are required to clarify it.

Although a substantial heterogeneity was detected in many pooled analyses, it did not have a significant impact on the results of these analyses, indicating that most evidences from our study should be considered to be stable and convincing. However, our study still had several potential limitations, and some cautions should be applied when interpreting these results. First, previous studies have shown that the presence of survivin rs9904341 was correlated in many cancer cell lines with increased survivin expression at both mRNA and protein levels when compared to normal cell line controls, indicating that transcriptional deregulation caused by this mutation in the promoter region of survivin gene might be an important mechanism involved in the aberrant expression of survivin in some cancers [52]. However, our study has included various types of cancer, and different cancers might have different characteristics while sharing the same mutation mechanism across these different cancers that might introduce a natural bias into our study. As a result, more convincing evidence from mutation cell-culturing studies of all above mentioned survivin SNPs in all these cancers is still required to draw a more solid conclusion. Second, a substantial heterogeneity was detected in the meta-analysis of survivin rs9904341. Through subgroup analyses stratified by ethnicity and tumor types, the heterogeneity obviously reduced, indicating that differences in ethnicity and tumor types might be the major contributor to heterogeneity. Third, our results were based on unadjusted estimates, while a more precise analysis should be conducted according to potentially confounding factors, such as age and gender. Fourth, most included studies of this meta-analysis were hospital-based, and thus the controls might not be representative of the general population, which might introduce some inevitable selection bias into our results. Finally, although no significant publication bias was detected, our meta-analysis included studies published only in English and Chinese, while papers written in other languages might be missed, which also possibly biased our results.

In conclusion, our study revealed that the survivin rs9904341 most likely contributed to increased susceptibility to tumor in Asians as well as to gastric, colorectal and bladder cancers. As for rs17878467, the T allele might be a protective factor for tumor, especially in Asians. Moreover, the survivin rs8073069 and rs2071214 seemed to be associated with an increased tumor risk in Asians, while there was no association between the survivin rs1042489 and tumor risk. However, further studies of high quality with larger sample sizes are still needed to confirm these findings.

## Supporting Information

Checklist S1 **PRISMA Checklist.**
(DOC)

Table S1 **Sensitivity analysis for heterogeneity.**
(DOCX)
