# Supplementary material for: Association of Survivin Polymorphisms with Tumor Susceptibility: A Meta-Analysis
Source: PLoS One. 2013 Sep 30;8(9):e74778. doi: 10.1371/journal.pone.0074778 (PMC3787000; doi:10.1371/journal.pone.0074778)
Supplement: Table S1 — Sensitivity analysis for heterogeneity. (DOCX) [file pone.0074778.s002.docx]

| **Table S1: Sensitivity analysis for** **heterogeneity** | | | | |
| --- | --- | --- | --- | --- |
|  |  | |  |  |
| ***Survivin***  **rs8073069** | **Study majorly contributed to the heterogeneity** | **Heterogeneity** **after excluding the study** | | **Pooled ORs of the remaining studies** |
|  |  | **P_h_** | ***I^2^*,%** | **OR 95% CI, P** |
| **Total/Ethnicity (Asian)** | | | | |
| C vs. G | Yang et al.’s study | 0.715 | 0.0 | 0.97 (0.82, 1.13), 0.667 |
| **C/C vs. G/G** | **Jang et al.’s study** | **0.291** | **10.4** | **2.00 (1.25, 3.19), 0.004** |
| Dominant genetic model | Yang et al.’s study | 0.969 | 0.0 | 0.91 (0.74, 1.11), 0.357 |
| ***Survivin***  **rs9904341** | **Study majorly contributed to the heterogeneity** | **Heterogeneity** **after excluding the study** | | **Pooled ORs of the remaining studies** |
|  |  | **P_h_** | ***I^2^*,%** | **OR 95% CI, P** |
| **Total** | | | | |
| **C vs. G** | **Jaiswal et al.’s study** | **0.001** | **80.1** | **1.18 (1.05, 1.33), 0.007** |
| C/C vs. G/G | Weng et al.’s study | 0.001 | 70.6 | 1.47 (1.19, 1.81), 0.001 |
| G/C vs. G/G | Wang et al.’s study | 0.001 | 56.5 | 1.05 (0.92, 1.20), 0.499 |
| Dominant genetic model | Radojevic-Skodric et al.’s study | 0.001 | 69.4 | 1.22 (1.06, 1.42), 0.008 |
| Recessive genetic model | Weng et al.’s study | 0.001 | 62.8 | 1.39 (1.19, 1.63), 0.001 |
| **Ethnicity** | | | | |
| **Caucasian** | | | | |
| C vs. G | Radojevic-Skodric et al.’s study | 0.001 | 80.9 | 1.12(0.84, 1.50), 0.429 |
| C/C vs. G/G | Radojevic-Skodric et al.’s study | 0.001 | 72.9 | 1.22(0.72, 2.07), 0.450 |
| G/C vs. G/G | Radojevic-Skodric et al.’s study | 0.001 | 72.6 | 1.10(0.75, 1.60), 0.638 |
| Dominant genetic model | Radojevic-Skodric et al.’s study | 0.001 | 78.8 | 1.15(0.77, 1.72), 0.504 |
| Recessive genetic model | Gazouli et al.’s study | 0.107 | 42.6 | 1.05(0.80, 1.38), 0.740 |
| **Asian** | | | | |
| **C vs. G** | **Jaiswal et al.’s study** | **0.001** | **79.7** | **1.24 (1.09, 1.41), 0.001** |
| C/C vs. G/G | Cheng et al.’s study | 0.001 | 74.2 | 1.43 (1.13, 1.80), 0.003 |
| G/C vs. G/G | Wang et al.’s study | 0.153 | 26.9 | 1.04 (0.95, 1.14), 0.365 |
| Dominant genetic model | Wang et al.’s study | 0.003 | 55.9 | 1.18 (1.02, 1.36), 0.024 |
| Recessive genetic model | Weng et al.’s study | 0.001 | 66.4 | 1.47 (1.23, 1.75), 0.001 |
| **Types of tumor** | | | | |
| **Gastric** | | | | |
| **C vs. G** | **Cheng et al.’s study** | **0.230** | **32.0** | **1.25 (1.03, 1.51), 0.023** |
| C/C vs. G/G | Cheng et al.’s study | 0.335 | 8.5 | 1.58 (1.07, 2.33), 0.022 |
| G/C vs. G/G | Borges et al.’s study | 0.087 | 59.0 | 1.46 (0.85, 2.49), 0.168 |
| Dominant genetic model | Cheng et al.’s study | 0.090 | 58.5 | 1.22 (0.72, 2.05), 0.466 |
| Recessive genetic model | Cheng et al.’s study | 0.911 | 0.0 | 1.44 (1.05, 1.97), 0.022 |
| **Colorectal** | | | | |
| C/C vs. G/G | Gazouli et al.’s study | 0.239 | 27.7 | 1.62 (1.23, 2.12), 0.001 |
| Recessive genetic model | Antonacopoulou et al.’s study | 0.187 | 42.6 | 1.76 (1.46, 2.13), 0.001 |
| **Other** | | | | |
| **C vs. G** | **Radojevic-Skodric et al.’s study** | **0.001** | **79.9** | **1.22 (1.03, 1.44), 0.024** |
| **C/C vs. G/G** | **Weng et al.’s study** | **0.001** | **68.6** | **1.48 (1.08, 2.02), 0.015** |
| G/C vs. G/G | Radojevic-Skodric et al.’s study | 0.001 | 71.3 | 1.16 (0.91, 1.48), 0.228 |
| Dominant genetic model | Radojevic-Skodric et al.’s study | 0.001 | 77.3 | 1.25 (0.97, 1.61), 0.080 |
| Recessive genetic model | Weng et al.’s study | 0.103 | 37.1 | 1.42 (1.26, 1.59), 0.001 |
| ***Survivin***  **rs2071214** | **Study majorly contributed to the heterogeneity** | **Heterogeneity** **after excluding the study** | | **Pooled ORs of the remaining studies** |
|  |  | **P_h_** | ***I^2^*,%** | **OR 95% CI, P** |
| **Total** | | | | |
| **G vs. A** | **Kawata et al.’s study** | **0.263** | **24.7** | **1.16 (1.01, 1.33), 0.042** |
| Dominant genetic model | Kawata et al.’s study | 0.184 | 38.0 | 1.10 (0.93, 1.30), 0.260 |
| **Ethnicity** | | | | |
| **Asian** | | | | |
| **G vs. A** | **Kawata et al.’s study** | **0.277** | **22.2** | **1.17 (1.02, 1.35), 0.029** |
| Dominant genetic model | Kawata et al.’s study | 0.234 | 31.1 | 1.12 (0.95, 1.33), 0.179 |
| ***Survivin***  **rs1042489** | **Study majorly contributed to the heterogeneity** | **Heterogeneity** **after excluding the study** | | **Pooled ORs of the remaining studies** |
|  |  | **P_h_** | ***I^2^*,%** | **OR 95% CI, P** |
| **Total/Ethnicity (Asian)** | | | | |
| T vs. C | Li et al.’s study | 0.017 | 75.5 | 1.23 (0.97, 1.56), 0.094 |
| T/T vs. C/C | Li et al.’s study | 0.015 | 76.2 | 1.49 (0.91, 2.43), 0.115 |
| Dominant genetic model | Weng et al.’s study | 0.066 | 63.3 | 1.05 (0.69, 1.60), 0.820 |
| Recessive genetic model | Weng et al.’s study | 0.112 | 54.4 | 1.03 (0.85, 1.25), 0.752 |
| P_h_: the P-value of heterogeneity; OR: odds ratio; CI: confidence interval. | | | | |
